# Radiation-induced short-range order in ceramics

**DOI:** 10.1093/jmicro/dfaf053

**Published:** 2025-12-02

**Authors:** Manabu Ishimaru

**Affiliations:** Department of Materials Science and Engineering, Kyushu Institute of Technology, Tobata, Kitakyushu, Fukuoka 804-8550, Japan

**Keywords:** order-to-disorder phase transformation, amorphization, short-range order, diffuse scattering

## Abstract

The development of radiation-tolerant materials is of technological importance for establishing safe operating systems in the nuclear industry, from power generation to the immobilization of high-level radioactive waste. Harsh radiation environments generate interstitials and vacancies in materials, and their accumulation leads to structural changes, including order-to-disorder phase transformations and amorphization. These structural changes are induced locally on an atomic scale; therefore, transmission electron microscopy is a useful technique for analyzing radiation effects in materials. In addition, the strong interaction between matter and electrons enables the detection of weak signals associated with phase transformations, such as diffuse scattering and halo rings. This article provides an overview of radiation-induced amorphous structures in materials consisting of light elements, such as boron carbide and silicon oxycarbide, as well as the short-range ordered structure that appears during an order-to-disorder phase transformation in fluorite structural derivatives.

## Teaser

Understanding the structural changes of materials in radiation environments is important for establishing safe operating systems in the nuclear industry. This article provides an overview of radiation-induced order-to-disordered phase transformation and amorphization in ceramics, analyzed using diffuse scattering and halo rings in electron diffraction patterns.

## Introduction

The temperature change over the past 50 years makes it clear that global warming is a worldwide problem [1]. To address this issue, the International Energy Agency has set a goal of achieving net-zero energy-related emissions by 2050 [2]. To reach this objective, the Japanese government set an interim target in April 2021 to reduce CO_2_ gas emissions by 46% in 2030 compared to the 2013 level [3]. Furthermore, the Seventh Strategic Energy Plan, approved by the Cabinet in February 2025, provides an outlook for the energy supply and demand in 2040 [4]. The power generation mix in 2022 and 2040 is summarized in Table 1 [5]. The proportion of thermal power generation will decrease significantly in the future. Conversely, the proportion of energy supplied by renewable sources is expected to increase from 22% in 2022 to 40–50% in 2040. However, renewable energies face challenges such as the difficulty of providing a stable power supply, limited installation sites and relatively high generation costs.

**Table 1.** Power system configuration in Japan in 2021, alongside prospective values for 2040 under the Seventh Strategic Energy Plan

Year	2022	2040
Renewable energy	22%	40–50%
Thermal power plant	72%	30–40%
Nuclear power plant	6%	∼20%

Nuclear power generation is considered an important alternative to thermal power generation as it provides a stable, clean electricity supply. However, accidents at nuclear power plants can have serious consequences. The disposal of high-level radioactive waste is also a global issue. To establish the safe and effective operation of the nuclear power plants, new structural and functional nuclear materials are required. The nuclear materials are exposed to harsh radiation environments, and therefore they are required to have not only thermal and chemical stability but also radiation tolerance. For this reason, the irradiation effects on various materials, ranging from metals to insulators, have been extensively investigated so far. Among the various materials, ceramics are currently used for nuclear fuels and cladding materials. They are also anticipated to be used for nuclear waste storage.

Nuclear fission reactions and radioactive decay produce energetic particles, including α-rays, β-rays, γ-rays, neutrons and fission fragments. These particles cause atomic-level damage to materials, such as interstitials and vacancies. The accumulation of this damage disrupts atomic configurations, resulting in an order-to-disorder phase transformation and amorphization. To develop radiation-tolerant materials, knowledge of radiation-induced structural changes is required. Various methods are used to evaluate irradiation-induced disordering processes. In diffraction techniques, information about the short-range order associated with disordering is obtained through diffuse scattering. However, diffuse scattering is much weaker than Bragg reflections and is widely distributed throughout the reciprocal lattice space, making it difficult to detect. Thanks to the strong interaction between electrons and matter, electron diffraction can easily detect weak signals. An extremely narrow beam, such as a nanobeam or an angstrom beam generated by a field emission gun, is also useful for analyzing disordered structures on an atomic scale [6–12].

**Fig. 1.**
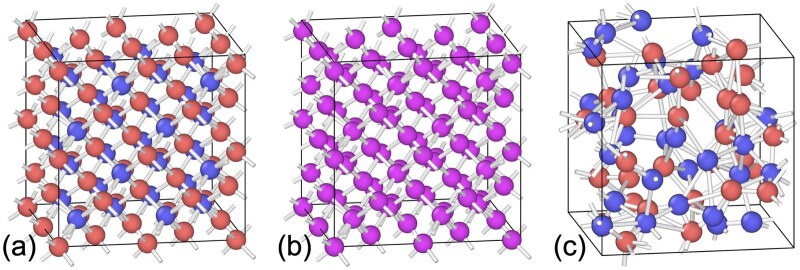
The atomic arrangement of the binary A_1-__*x*_B_*x*_ alloy: (a) a perfectly ordered crystalline structure, (b) a perfectly disordered crystalline structure and (c) an amorphous structure. In (a), the A (red) and B (blue) atoms occupy specific crystal lattice sites. In (b), however, they occupy positions randomly, with probabilities corresponding to their composition, and no distinction can be made between atomic sites. In (c), the crystal lattice has collapsed geometrically.

This review focused on the irradiation-induced short-range order in ceramics. The term ‘short-range order’ is used in two senses here, as illustrated in Fig. 1. Figure 1a shows the atomic arrangement of the ordered binary alloy A_1-__*x*_B_*x*_, in which the A and B atoms occupy specific crystal lattice sites. In a perfectly disordered state, the atoms occupy these sites according to their respective probabilities, as shown in Fig. 1b. In real crystals, a completely disordered state is rare, and some degree of order, known as chemical short-range order, exists. Another type of short-range order is amorphous, where the crystal lattice has collapsed geometrically (Fig. 1c). In this case, both topological and chemical short-range orders must be taken into account.

## Irradiation-induced amorphous structure of boron carbide

Due to its excellent material properties, boron carbide (B_4_C) is used for structural components and electronic devices. Its high neutron absorption cross-section makes it useful for neutron absorbers. Although the amorphization mechanism of B_4_C in radiation environments has been extensively studied [13–16], it remains unclear due to limited knowledge of amorphous structures. The probability of finding other atoms within a distance ranging from *r* to *r* + *dr* around a specific atom can be used to evaluate the structure of an amorphous material. These are known as radial distribution functions and atomic pair distribution functions [17], and they can be obtained via diffraction techniques. However, for B_4_C, this information is difficult to obtain via X-ray diffraction because of the small atomic scattering factors of its constituent elements. Electron beams scatter due to the Coulomb potential, and their interaction with matter is 10^3^–10^4^ times greater than that of the X-rays. Because of this, we investigated the amorphous structure of B_4_C using electron diffraction [18].

**Fig. 2.**
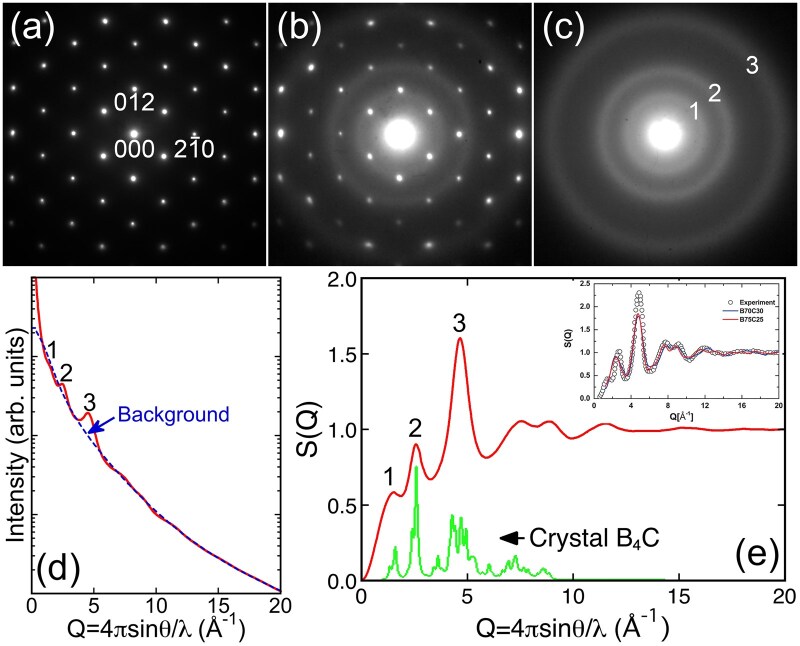
(a–c) Change in electron diffraction pattern with damage accumulation: (a) unirradiated, (b) medium damaged, and (c) maximum damaged regions, which were reproduced from Ref. [18]. The pattern of (a) corresponds to the [$12\overset{-}{1}$] zone-axis of B_4_C. Both the Bragg spots and halo rings appear in (b), and complete amorphization occurs at the maximum damaged region in (c). The numbers in (c) correspond to the first, second and third rings. (d) An intensity profile of the halo diffraction pattern as a function of the scattering vector (*Q*). (e) An interference function, *S*(*Q*), of amorphous B_4_C and the calculated intensity profile of crystalline B_4_C. The inset is the *S*(*Q*) obtained by neutron diffraction (circles) and *ab initio* molecular-dynamics simulation (lines) of amorphous boron carbide, which was reproduced from Ref. [27].

Polycrystalline B_4_C pellets were irradiated with 2 MeV Au ions at room temperature to a fluence of 5×10^15^ cm^−2^. According to Monte Carlo simulations using the SRIM (Stopping and Range of Ions in Matter) code [19], the maximum concentration of Au was estimated to be ∼0.4 at.% [18]; therefore, the effects of projectiles on structural analysis are negligible. The SRIM calculations also revealed that damage is maximized at ∼300 nm [18]. Cross-sectional TEM observations were performed to examine structural change as a function of depth. Figure 2a–c shows the change in the electron diffraction pattern with damage accumulation, obtained from the same grain at different depths using an electron beam with a diameter of ∼80 nm. The depths from the surface are >500 nm (well beyond the end of the range (Fig. 2a), ∼150 nm (Fig. 2b) and ∼300 nm (Fig. 2c). The pattern of Fig. 2a obtained from the unirradiated region was consistent with the [$12\overset{-}{1}$] zone-axis of crystalline B_4_C (space group: $R\overset{-}{3}m$ (No. 166); *a *= 5.6043 Å, *c *= 12.0841 Å [20]). As damage accumulates, halo rings that overlap the Bragg spots appear in Fig. 2b. Ultimately, the region with maximum damage underwent complete amorphization. Figure 2d shows the intensity profile of the halo ring obtained by integrating the intensity over the entire ring. The scattering vector (*Q*) is defined as 4πsinθ/λ, where θ and λ are the half-scattering angle and the electron wavelength, respectively. The numbers correspond to the position of the halo ring indicated in Fig. 2c. The amorphous structure of B_4_C was previously analyzed using electron diffraction, but the available intensity profile is limited to *Q *< 10 Å^−1^ [21]. In contrast, the present study observed intensity oscillations even with *Q *> 10 Å^−1^.

Subtracting the background from the averaged intensity, <*I*(*Q*)>, yielded the interference function, $S(Q)=\frac{<I(Q)>-{N<f}^{2}(Q)>}{{N<f(Q)>}^{2}}$, as shown in Fig. 2e. Here, *N* is the number of atoms, *f*(*Q*) is the atomic scattering factor for electrons, <*f*(*Q*)>^2^ is the square of the averaged atomic scattering factor and <*f*^2^(*Q*)> is the average of the squared atomic scattering factor [17]. The details of the radial distribution analysis based on electron diffraction are described elsewhere [22–24]. For comparison, the intensity profile of crystalline B_4_C is also indicated. A very weak intensity profile was recorded well up to ∼20 Å^−1^. As shown in the inset, this intensity profile is comparable to those obtained by neutron diffraction [25] and *ab initio* molecular-dynamics simulation [26, 27], suggesting the validity of the present study. Thanks to the strong interaction between matter and electrons, we successfully obtained an intensity profile equivalent to that of neutron diffraction in <1 s of exposure time, indicating that electron diffraction is a powerful technique for analyzing amorphous materials.

**Fig. 3.**
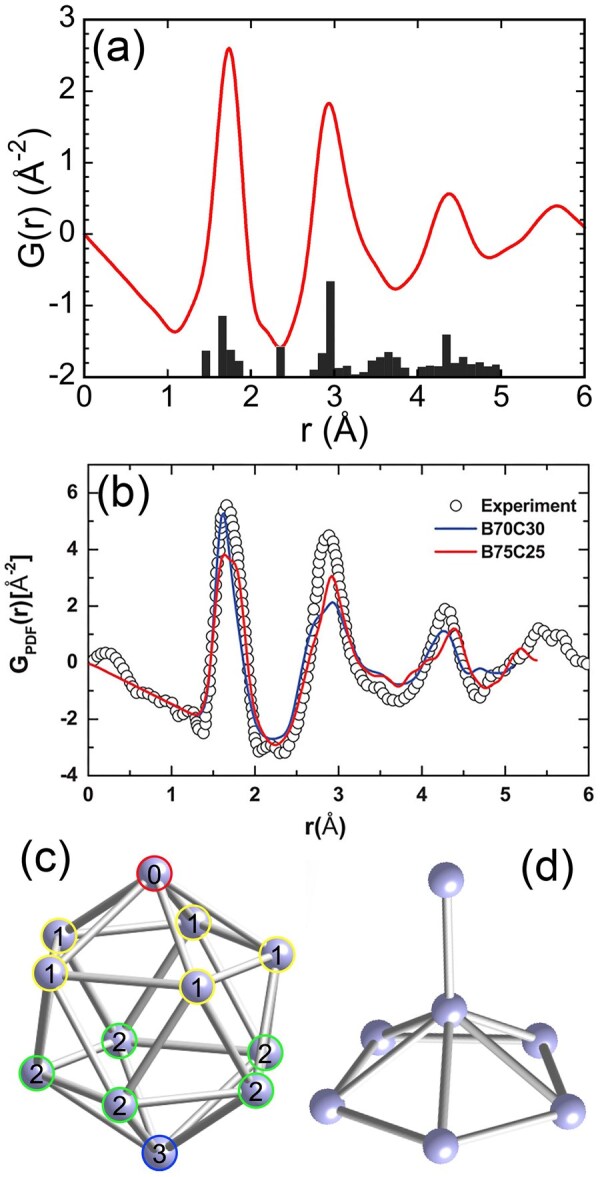
(a) Reduced radial distribution function, *G*(*r*), of amorphous B_4_C. For comparison, the interatomic distances in crystalline B_4_C are indicated as the bar with a width of 0.1 Å on the horizontal axis. Prominent peaks corresponding to the first and second nearest neighbors are present at ∼1.7 and ∼2.9 Å, whereas there is no peak at ∼3.6 Å corresponding to the third nearest neighbor of crystal B_4_C. (b) The *G*(*r*) obtained by neutron diffraction (circles) and *ab initio* molecular-dynamics simulation (lines) of amorphous boron carbide, which was reproduced from Ref. [27]. It is apparent that the *G*(*r*) of (a) is in agreement with that obtained by neutron diffraction and *ab initio* molecular-dynamics simulation. (c) A schematic diagram of an icosahedral cluster, which is a constituent element of the B_4_C crystal structure. For simplicity, boron and carbon atoms are not distinguished. The numbers correspond to the *n*-th nearest neighbor from the atom indicated by ‘0’. (d) A pentagonal pyramid, which is a favorable structural unit in the amorphous B_4_C network.

By performing a Fourier transform on the *S*(*Q*), the reduced radial distribution function $G(r)=\frac{\pi }{2}\int_{0}^{\infty }QS(Q)\sin\left(Qr\right)dQ$, was obtained in Fig. 3a. For comparison, the interatomic distance in the crystal structure of B_4_C is also indicated. Prominent peaks appeared at 1.7 and 2.9 Å, corresponding to short-range order. The *G*(*r*) was similar to that of amorphous B_2.5_C obtained by neutron diffraction (circle) [25] and B_70_C_30_ (blue) and B_75_C_25_ (red) constructed by *ab initio* molecular-dynamics simulation [27] (Fig. 3b). The crystal structure of B_4_C consists of icosahedral clusters [28–30]. Figure 3c shows an icosahedral cluster, in which the numbers of ‘1’, ‘2’ and ‘3’ correspond, respectively, to the first, second and third nearest neighbors of the atom indicated by ‘0’. Peaks corresponding to the first and second nearest neighbors were present, but there was no peak corresponding to the third nearest neighbor. This result suggested that the pentagonal pyramid shown in Fig. 3d is a favorable structural unit in the amorphous B_4_C network rather than the icosahedron. In fact, the pentagonal pyramid was observed in amorphous B_4_C generated by *ab initio* molecular-dynamics simulations [26].

## Impact of amorphous structure on radiation tolerance of silicon oxycarbide

Amorphization causes volume swelling and microcracks, which degrade the mechanical properties of materials. To avoid the negative effects of irradiation-induced amorphization, amorphous materials are being considered for use in nuclear reactor components. Metallic glasses have attracted considerable attention for their application in extreme environments due to their high mechanical strength, high fracture toughness and good corrosion resistance [31]. However, they easily crystallize under irradiation environments [32–36]. Recent studies have revealed that although amorphous structures are more radiation-resistant in amorphous high-entropy alloys, they still crystallize under high-temperature irradiation conditions [37].

**Fig. 4.**
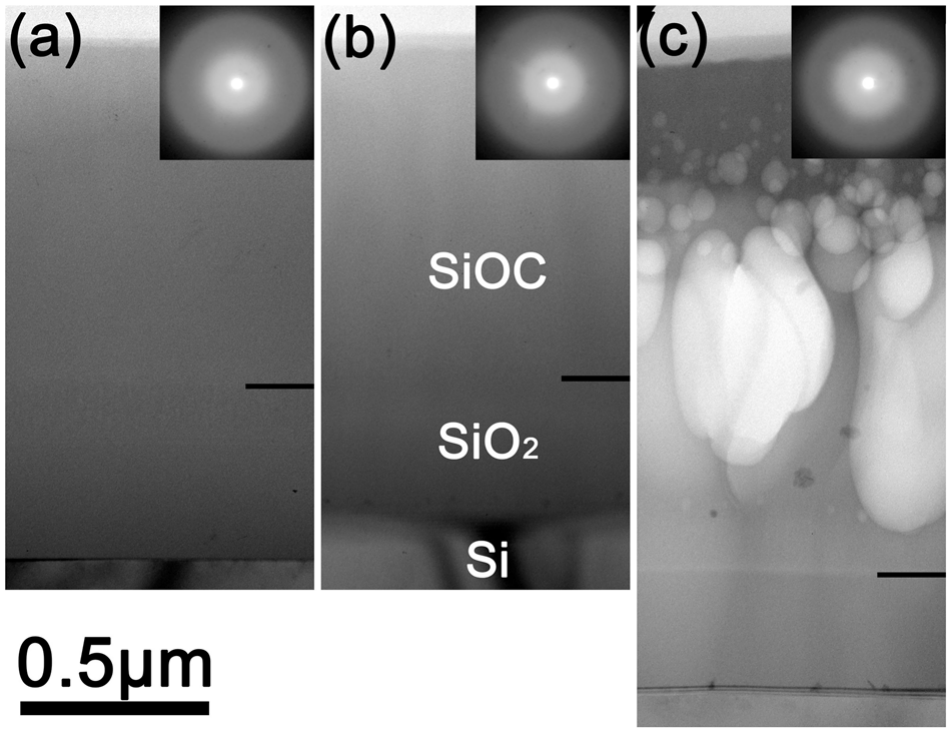
Cross-sectional TEM images of He ion irradiated SiOC with different compositions, which were reproduced from Ref. [40]. The ratio of SiO_2_ to SiC is as follows: (a) 2:1, (b) 1:1, and (c) 1:2. The interface between the SiOC and SiO_2_ is indicated by a line. Electron diffraction patterns taken from the maximum damage region reveal that the amorphous structure is maintained. No significant structural changes occur in (a) and (b), whereas He bubbles and the accompanying volume swelling are induced in (c).

Amorphous silicon oxycarbide (SiOC) has a quite high compressive strength and retains its amorphous structure up to 1200°C [38]. Furthermore, no helium (He) bubbles or volume swelling were observed under irradiation conditions corresponding to 113 at.% He ion implantation [39]. (Note that this concentration was estimated using Monte Carlo simulations, which do not account for atomic diffusion.) To clarify the origin of He bubble suppression in SiOC, we investigated its amorphous structure before and after irradiation using atomic pair-distribution analysis [39–41]. The SiOC thin films deposited on SiO_2_/Si substrate were irradiated with 120 keV He ions to a fluence of ∼10^18^ He/cm^2^ at room temperature. According to the Monte Carlo simulations using the SRIM-2008 software [19], the maximum concentration of He was estimated to be ∼90 at.% at a depth of ∼800 nm [40]. Figure 4 shows cross-sectional TEM images of 120 keV He ion irradiated SiOC thin film deposited on SiO_2_/Si substrate. The ratio of SiO_2_:SiC is (i) 2:1 (SiO_2_-rich), (ii) 1:1 (equiatomic) and (iii) 1:2 (SiC-rich). The SiO_2_-rich (Fig. 4a) and equiatomic SiOC (Fig. 4b) exhibited uniform contrast, and no significant changes were observed. In contrast, large He bubbles formed in the SiC-rich SiOC (Fig. 4c), resulting in a significant volume swelling. No Bragg spots were observed in the electron diffraction pattern shown as the inset, suggesting that the radiation-induced crystallization was highly suppressed in SiOC.

**Fig. 5.**
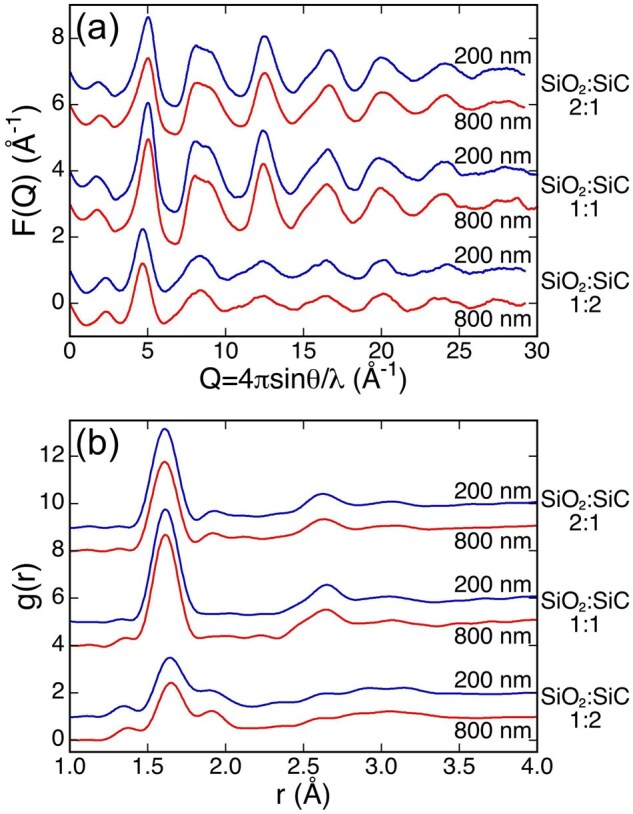
(a) Reduced interference functions, *F*(*Q*), of He ion irradiated SiOC with different concentrations. Electron diffraction patterns were recorded at depths of 200 nm and 800 nm, where the concentration of implanted He is negligible and maximum, respectively. (b) Atomic pair-distribution functions, *g*(*r*), obtained by Fourier transforming the *F*(*Q*) in (a), which was reproduced from Ref. [40]. No significant differences were observed in the *g*(*r*) obtained from different depths. The profile of the SiC-rich SiOC is quite different from that of other SiOCs.

To investigate the effects of He implantation on amorphous structures, electron diffraction patterns were acquired at depths of 200 and 800 nm, where the concentration of implanted He is negligible and maximum, respectively. Figure 5a shows the reduced interference function, *F*(*Q*) = *QS*(*Q*), of SiOC with different compositions. Intensity oscillations were observed up to a scattering vector of *Q* = ∼30 Å^−1^, which is much larger than the value (*Q *= 17 Å^−1^) obtained by X-ray diffraction of amorphous SiOC [42]. This suggested that a precise structural analysis is feasible in the present study. The *F*(*Q*) was similar for the SiO_2_-rich and equiatomic samples but different for the SiO_2_-poor samples. The atomic pair-distribution functions, $g(r)=\frac{G(r)}{4\pi r\rho_{0}}+1$, were obtained by Fourier transforming the *F*(*Q*). Here, *ρ*_0_ is the average number density. The resulting *g*(*r*) is shown in Fig. 5b. Because the height of the peak is strongly affected by multiple scattering depending on the thickness of the sample in the electron beam direction, only the peak position in *g*(*r*) was discussed here. The *g*(*r*) of the SiO_2_-rich and equiatomic samples was similar to that of SiO_2_ [40]: prominent peaks were present at ∼1.6, ∼2.6 and ∼3.1 Å, which were assigned to the first nearest neighbor of Si–O and the second nearest neighbors of O–O and Si–Si, respectively. In contrast, a significant change in the amorphous structure was observed in the SiC-rich SiOC sample: the Si–O and O–O peaks diminished, and a peak due to Si–C atomic pairs emerged at ∼1.9 Å [39, 43, 44].

No significant changes were observed in the short-range ordered structure associated with damage accumulation in SiOC, as shown in Fig. 5b. Therefore, the excellent resistance of amorphous SiOC to He bubble formation may be related to its medium-range ordered structure. It has been reported that the first peak in *F*(*Q*) of SiO_2_ appears at ∼1.5 Å^−1^, which corresponds to the atomic correlation of ∼4 Å in real lattice space. This first peak is known as the ‘first sharp diffraction peak (FSDP)’ and reflects the medium-range order in amorphous networks. The SiOC with an equal atomic composition and a SiO_2_-rich composition had a first peak located at ∼1.5 Å^−1^, which is almost the same position as the SiO_2_ peak. The origin of the FSDP remains a subject of debate [45–49], but a correlation has been observed between its scattering vector, *Q*_FSDP_, and the size of the ring (or network void) formed by the SiO_4_ tetrahedra. Based on the value of *Q*_FSDP_ in Fig. 4a, the diameter of the network void was estimated to be 3.5–3.7 Å, which is much larger than the diameter of a He atom (0.62 Å). This suggests that He atoms are rapidly ejected from the implanted region in the SiO_2_-rich and equiatomic specimens, which suppresses the formation of He bubbles. The diameter of the network voids in SiC-rich SiOC is still larger than that of a He atom. However, the diffusion rate of He is expected to decrease as the diameter of the network voids reduces. Consequently, He accumulation in the implanted region is more pronounced in SiC-rich SiOC, resulting in He bubble formation.

## Order-to-disordered phase transformation in complex oxides

**Fig. 6.**
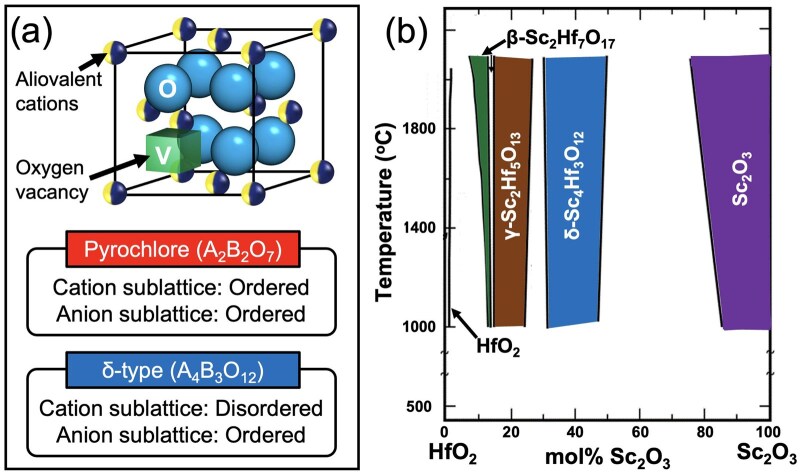
(a) Atomic arrangement of the oxygen-deficient fluorite structure. Cations with different charges occupy the cation sublattice and oxygen vacancies are introduced to ensure electrical neutrality. While both the cation and anion sublattices exhibit atomic ordering in the pyrochlore structure, only the oxygen vacancies are ordered in the δ-type structure. (b) A phase diagram of the Sc_2_O_3_-HfO_2_ pseudo-binary system [78]. Various compounds, so called the fluorite structural derivatives, are present.

High-level radioactive waste is stored in borosilicate glass containers for geological disposal. However, the lifetime of the glass containers is much shorter than the half-life of radioactive elements. Crystalline ceramics are anticipated as a new container material because of their excellent properties, such as thermal stability and chemical durability [50–53]. They can also incorporate minor actinides into their lattice structure. Host cations are often substituted with aliovalent cations to improve functionality, such as thermal and electronic conductivity, which creates a deficiency on the anion sublattice in order to maintain electrical neutrality. Oxide ceramics with a fluorite-type structure, where oxygen anions form a face-centered cubic lattice and metal cations occupy its tetrahedral interstitial sites, exhibit excellent radiation tolerance [54–57]. Therefore, the radiation behavior of oxygen-deficient fluorite ceramics (Fig. 6a), in which oxygen vacancies are introduced to maintain charge neutrality in the presence of aliovalent cations, is also investigated. When these oxygen vacancies are arranged regularly, ordered phases called fluorite structural derivatives form. Depending on the cation radius ratio and composition, various fluorite structural derivatives are formed. Ceramics with a pyrochlore structure are expressed as A_2_B_2_O_7_, where A and B represent trivalent and quadrivalent cations, respectively. This structure is stabilized by an ionic radius ratio of *r*_A_/*r*_B_ between 1.46 and 1.78 [58]. The structure changes to a defect fluorite structure when *r*_A_/*r*_B_ is <1.46.

Much effort has been devoted to predicting the radiation tolerance of the fluorite structural derivatives [59–67]. Since cation antisite defects increase the energy of the system, the cation radius ratio can be used as a guideline for predicting crystal lattice stability [68] and the amorphization resistance [53, 69]. A phase transformation from ordered pyrochlore to disordered fluorite is often observed prior to amorphization, and therefore the disordering process of pyrochlore ceramics due to damage accumulation has also been studied. In the pyrochlore structure, atomic ordering occurs in both cation and anion sublattices. Recent studies of the order-to-disorder phase transformation using neutron total scattering experiments with pair distribution function analysis have revealed that the long-range ordered pyrochlore structure transforms into the short-range ordered weberite-type structure [70–75]. This means the disordering process is more complex than we ever thought.

The Sc_2_O_3_-ZrO_2_ [76, 77] and Sc_2_O_3_-HfO_2_ [78] systems have several fluorite structural derivatives with different compositions. Figure 6b shows the phase diagram of the Sc_2_O_3_-HfO_2_ pseudo-binary system [78]. The fluorite structural derivatives, including β-Sc_2_Hf_7_O_17_, γ-Sc_2_Hf_5_O_13_ and δ-Sc_4_Hf_3_O_12_, are present in the shaded area. Sc_2_O_3_ has the bixbyite structure, which is also a fluorite structural derivative. Bixbyite is one of the most common polymorphs found in sesquioxide (A_2_O_3_) compounds. It is characterized by a particular ordering of vacancies on the oxygen sublattice. Specifically, one-fourth of the oxygen sublattice sites are vacant in bixbyite, compared to one-seventh in the δ-phase. This comparison is referenced to the parent fluorite structure. The Sc_2_O_3_-ZrO_2_ system also has compounds with the same compositions as those in the Sc_2_O_3_-HfO_2_ system. Among these compounds, the δ-phase has the greatest radiation tolerance [79]. Unlike the pyrochlore structure, the cation sublattice is disordered in δ-Sc_4_Zr_3_O_12_ and partially disordered in δ-Sc_4_Hf_3_O_12_ (Fig. 6a). Thus, the δ-phase is an ideal model system for studying the structural changes in fluorite structural derivatives with long-range order of anion sublattice. Here, we focused on the anomalous short-range ordered structure observed in the ion-irradiated δ-Sc_4_Hf_3_O_12_ [80, 81]. The irradiation-induced structural changes in the β- and γ-phases have been reported elsewhere [82].

**Fig. 7.**
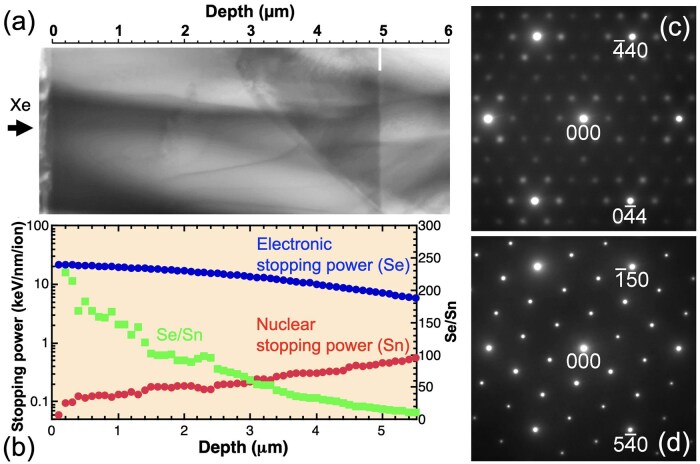
(a) Cross-sectional TEM image of 92 MeV Xe^26+^ ion irradiated δ-Sc_4_Hf_3_O_12_. The contrast changes at a depth of ∼5 μm. (b) Stopping powers as a function of depth, calculated using Monte Carlo simulations: nuclear stopping power (red circles), electronic stopping power (blue circles), and their ratio (green squares). The electronic stopping power is much larger than the nuclear stopping power, suggesting that ionization is dominant in the region displayed in (a). (c and d) Selected-area electron diffraction patterns taken from the dark contrast area on the surface side and the bright contrast area on the substrate side. These correspond to (c) the bixbyite structure with the [111] zone-axis and (d) the δ-type structure with [001] zone-axis. (a), (c) and (d) were reproduced from Ref. [81].

Figure 7a shows the cross-sectional TEM image of δ-Sc_4_Hf_3_O_12_ irradiated with 92 MeV Xe^26+^ ions to a fluence of 10^14^ cm^−2^ at room temperature. The ions were irradiated from the left side, and ‘0 μm’ represented the surface location. The contrast was found to change at a depth of 5 μm. Figure 7b shows the stopping power calculated by Monte Carlo simulations based on the SRIM code [19]. Projectile ions lose energy in two ways: through elastic collisions with target nuclei and through inelastic collisions, such as electronic excitation and ionization. These two processes are referred to as the nuclear stopping power (Sn) and electronic stopping power (Se), respectively. The ratio of Se/Sn was nearly equal to 20 at 5 μm, and the electronic stopping power was dominant in the region displayed in Fig. 7a. Selected-area electron diffraction patterns taken from the dark (the surface side) and bright (the substrate side) contrast regions are shown in Fig. 7c and d, respectively. In addition to the fundamental lattice spots, superlattice reflections were observed in Fig. 7d, which were attributed to the ordering of the oxygen vacancies. This pattern was consistent with the (001) reciprocal plane of the δ-phase, which is identical to that of the pristine structure. On the other hand, spotty diffuse scattering appeared in Fig. 7c and was located in a position different from that of the superlattice reflections in Fig. 7d. This pattern was found to correspond to the (111) reciprocal plane of the bixbyite phase. This suggested that the long-range ordered δ-phase transforms into the short-range ordered bixbyite phase. This structural change has also been observed in δ-Sc_4_Zr_3_O_12_ irradiated with 300 keV Kr^2+^ ions, where the knock-on effect is dominant [83].

**Fig. 8.**
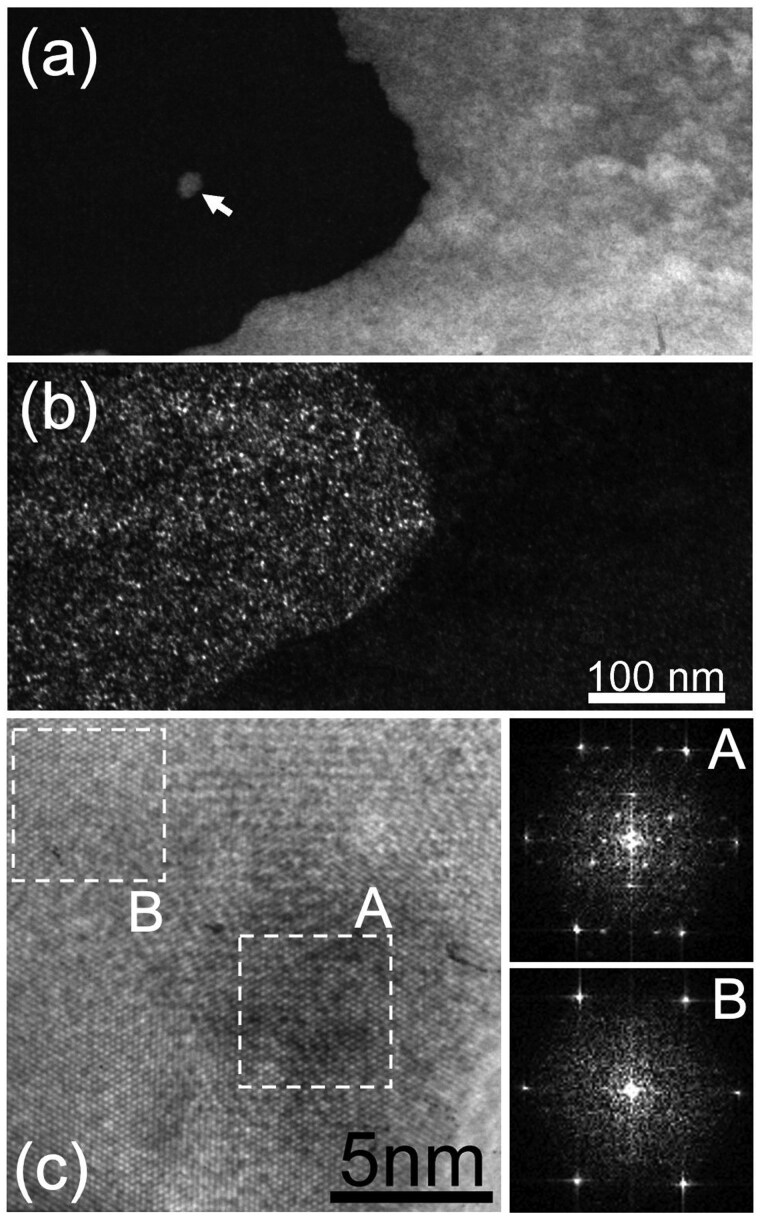
(a, b) Cross-section dark-field TEM images obtained from the boundary where the contrast changes. These were taken using (a) the superlattice spot due to the δ-type structure and (b) the diffuse spot due to the bixbyite structure. The ion beam was irradiated from the left-hand side of the image. The entire right-hand side of (a) exhibits bright contrast, suggesting that the δ-phase remains in a long-range ordered state. The bright contrast indicated by the arrow in (a) represents the region transformed into a δ-type structure by the convergent electron beam during beam alignment. In contrast, the bixbyite clusters are densely dispersed on the left-hand side of (b). (c) A high-resolution TEM image taken from the bixbyite region, along with fast Fourier transform diagrams of the square regions. The ordered bixbyite clusters are embedded in the disordered fluorite matrix. These images were reproduced from Ref. [81].

To examine the distribution of the bixbyite phase, dark-field TEM images were taken around the boundary of the contrast change. Figure 8a and b shows dark-field TEM images obtained by using the superlattice spot of the δ-structure and the diffuse spot of the bixbyite structure, respectively. The ion beam was irradiated from the left side, and this region was a single crystal [81]. A homogeneous bright contrast was observed in the substrate side in Fig. 8a, suggesting that the δ-phase keeps the long-range ordered state. Note that the bright region marked by the arrow is the δ-phase induced by electron beam irradiation, as described below. On the other hand, bright dots <10 nm were dispersed on the surface side (Fig. 8b). Figure 8c shows a high-resolution TEM image obtained from the bixbyite region. Dark dots appeared to be arranged regularly in Region A. Indeed, a high-resolution TEM image and their fast Fourier transform diagrams revealed that the bixbyite clusters are embedded in the fluorite matrix (Fig. 8c). That is, microdomains, which are small regions with a higher degree of order than the disordered matrix [84], were formed as short-range order.

**Fig. 9.**
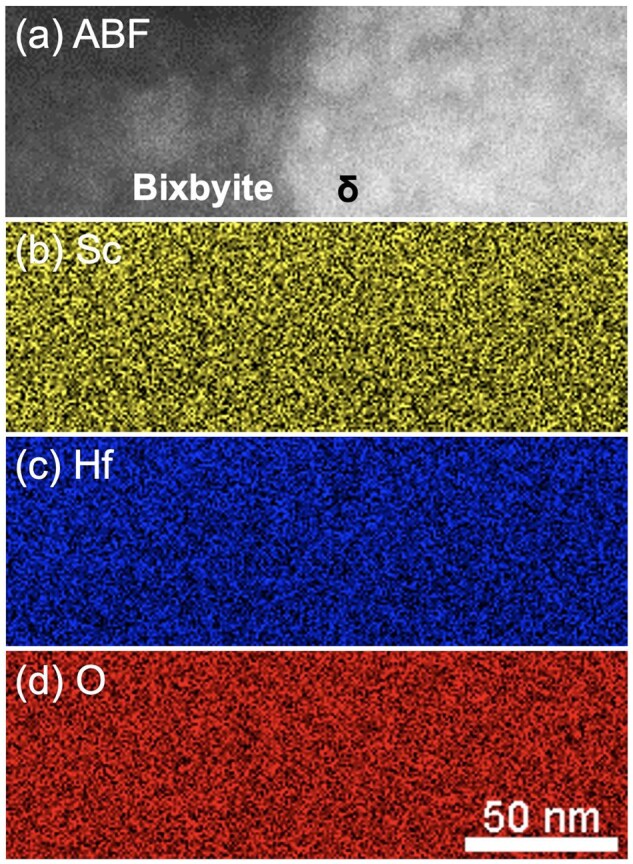
(a) Annular bright-field STEM image taken from the interface between the bixbyite and δ-phase regions. The ion beam was irradiated from the left-hand side of the image, and the dark and bright contrast regions are the bixbyite and δ-phases. (b–d) Elemental maps obtained by energy-dispersive X-ray spectroscopy: (b) Sc, (c) Hf and (d) O. There is no significant compositional change between the bixbyite and δ-phase regions, suggesting that the structural change from the δ to bixbyite phases is induced via a congruent phase transformation.

Of the compounds shown in the phase diagram in Fig. 6b, only Sc_2_O_3_ has a bixbyite structure. Its composition differs greatly from that of the δ-phase [85]. To investigate whether compositional changes accompany structural changes, elemental mapping was performed using energy-dispersive X-ray spectroscopy. Figure 9a shows an annular bright-field scanning TEM image of the interface between the bixbyite and δ regions, along with the distribution of (Fig. 9b) Sc, (Fig. 9c) Hf and (Fig. 9d) O. There were no significant compositional changes between the bixbyite and δ regions. This suggested that the δ-to-bixbyite structural change is due to a congruent phase transformation. In other words, the radiation-induced bixbyite phase had a similar cation composition to the δ-phase.

**Fig. 10.**
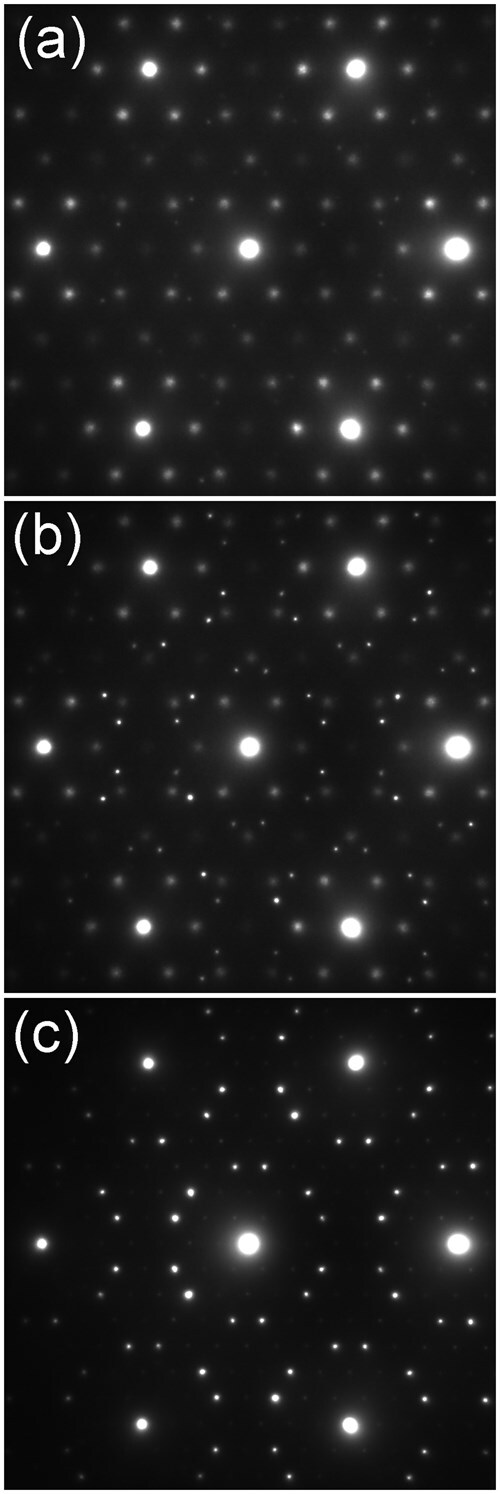
Changes in electron diffraction patterns during 200-keV electron beam irradiation: (a) 0 min, (b) 1 min and (c) 5 min. The diffuse spots due to the bixbyite phase in (a) become weak, and the sharp superlattice spots appear after electron beam irradiation. The pattern in (c) corresponds to the (001) diffraction pattern of the δ-type structure, which is composed of two different orientations. These diffraction patterns were reproduced from Ref. [81].

To confirm that compositional changes are not necessary for structural changes, we investigated the stability of the bixbyite phase under electron beam irradiation [81]. For electron beam irradiation experiments, we used the largest possible condenser aperture. Consequently, the flux was much greater than that of typical TEM observations. Figure 10 shows the evolution of the diffraction pattern during irradiation with a 200-keV electron beam at room temperature. The temperature rise during electron beam irradiation was estimated to be as low as 20 K [81]. The pattern of Fig. 10a corresponded to the bixbyite structure viewed along the [111] direction. The diffuse bixbyite spots became weaker with the electron beam irradiation, and new sharper spots appeared (Fig. 10b). The pattern of Fig. 10c could be interpreted by the overlapping of the δ-phase with the different variant [81]. Since electron beam irradiation was performed in a vacuum, a decrease in oxygen concentration was expected. There are fewer oxygen vacancies in the δ-phase than in the bixbyite phase. As described above, the δ (A_4_B_3_O_12_) and the bixbyite (A_2_O_3_) phases have one-seventh and one-fourth oxygen vacancies on the anion sublattice, respectively. However, the structural change from the bixbyite to the δ-phase was opposite to what was predicted from electron beam irradiation. It was also confirmed that bixbyite reverts to the δ*-*phase under thermal annealing at 300°C in an ambient atmosphere [81]. This suggested that the structural change observed here is not sensitive to the atmosphere.

The amount of energy transferred to an atom by a 200 keV electron beam is as follows: 11.7 eV for Sc, 2.9 eV for Hf and 32.8 eV for O. Decreasing the acceleration voltage was found to delay the transformation from the bixbyite to the δ-phase [81]. This suggested that the structural change induced by electron beam irradiation occurred as a knock-on effect. Assuming a threshold displacement energy of 20 eV (a typical value for ceramics), the bixbyite-to-δ phase transformation was induced by the rearrangement of oxygen vacancies. In other words, no significant compositional changes occurred during the electron beam-induced structural change, which was consistent with the congruent phase transformation. This was quite a different structural change predicted from the equilibrium phase diagram (Fig. 6b) in which the bixbyite and δ-phases were present at 80–100 mol.%Sc_2_O_3_ and 30–50 mol.% Sc_2_O_3_, respectively [78].

**Fig. 11.**
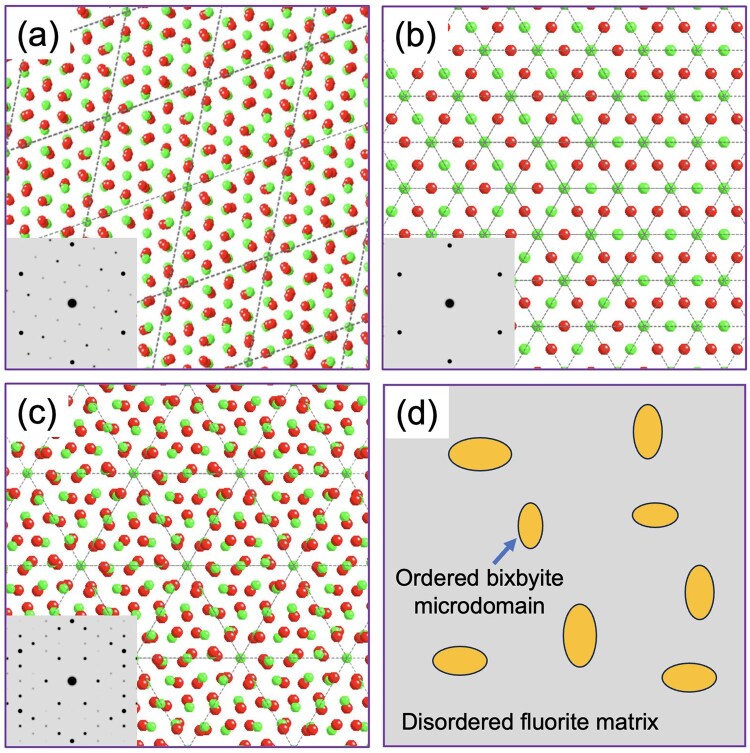
A summary of structural changes during the order-to-disorder phase transformation. (a) Atomic arrangements of (a) the rhombohedral ordered δ-type structure projected along the [001] direction, (b) the cubic disordered fluorite structure projected along the [111] direction and (c) the cubic ordered bixbyite structure projected along the [111] direction. Green and red circles denote cations and oxygen, respectively. The corresponding electron diffraction patterns are indicated in the insets. Irradiation induces cation anti-site defects and anion Frenkel pairs in the pristine structure (a) and accordingly disordering progresses. However, the degree of order does not decrease monotonically. Rather, an ordered phase with a different structure than the pristine form emerges locally within the damaged region. (d) Microdomain model in which the ordered bixbyite clusters are dispersed in the disordered fluorite matrix.

Figure 11 summarizes the structural changes in fluorite derivatives with accumulated damage. Previous studies have considered that the order-to-disordered phase transformation, i.e. the change from Fig. 11a to b, proceeds through the accumulation of cation anti-site defects and anion Frenkel pairs. (The cation sublattice is disordered or partially disordered in the δ-phase, but the general structural changes are described here.) This means that the degree of order of the pristine structure decreases monotonically with damage accumulation. On the other hand, bixbyite clusters were found in the damage region of ion-irradiated δ-Sc_4_Hf_3_O_12_; the short-range order could be described by microdomains, defined as small regions with a higher degree of order than that of the disordered matrix. Interestingly, these microdomains had a different structure, i.e. bixbyite, from the pristine δ-type structure. The formation of short-range order with other structures was also confirmed in pyrochlore (A_2_B_2_O_7_), which transforms to weberite (A_3_BO_7_) [70–75]. In contrast, the compositional change was found to be negligible in the transformation from the δ to bixbyite, suggesting that the structural change occurs via a congruent phase transformation.

## Concluding remarks and outlook

Accumulated damage to materials in radiation environments often induces order-to-disorder and crystal-to-amorphous phase transformations. Understanding these structural changes is crucial for predicting the reliability of materials used in the nuclear power industry. This article reviewed studies on radiation-induced short-range ordered structures in ceramics. Short-range order can be detected as diffuse scattering and halo rings in diffraction patterns, but these signals are very weak and broadly distributed in the reciprocal lattice space. Thanks to the strong interaction between matter and electrons, TEM and electron diffraction have successfully identified the structure of amorphous materials composed of light elements, as well as the sub-10-nanometer microdomains embedded within the disordered matrices.

This paper presents structural information obtained from electron diffraction patterns acquired over a wide area. Extreme damage regions, such as ion tracks and cascades, form along ion trajectories, and material degradation progresses due to their overlap. While these regions maintain crystallinity in radiation-resistant materials, metastable phases may occasionally form. These phases can be analyzed using high-resolution TEM images. Conversely, amorphization occurs in materials with low irradiation resistance; however, to our knowledge, there has been no direct structural analysis of the amorphous regions. The overwhelming advantage of using electron beams compared to X-rays and neutrons is the ability to achieve an extremely fine beam. By using nanobeam and angstrom-beam electron diffraction, amorphous structures locally produced by energetic ions can be identified.

This review has addressed structural changes induced by ion irradiation, but electron beam irradiation also alters the atomic arrangement through knock-on and electron excitation effects [86–90]. Electron beam irradiation can facilitate the formation of metastable phases and establish low-temperature synthesis techniques. A recent article in this journal explained how metastable phases form due to electronic excitation effects [91]. See that article for more information.

## Data Availability

Data will be made available on request.
